# The effect of aqueous extract of Xinjiang *Artemisia rupestris* L. (an influenza virus vaccine adjuvant) on enhancing immune responses and reducing antigen dose required for immunity

**DOI:** 10.1371/journal.pone.0183720

**Published:** 2017-08-25

**Authors:** Ailian Zhang, Danyang Wang, Jinyao Li, Feng Gao, Xucheng Fan

**Affiliations:** 1 Xinjiang Key Laboratory of Biological Resources and Genetic Engineering, College of Life Science and Technology, Xinjiang University, Urumqi, China; 2 Urumqi Center for Disease Control and Prevention, Urumqi, China; Taibah University, SAUDI ARABIA

## Abstract

Potent adjuvant can improve the effectiveness of vaccines and reduce the antigen doses required for initiating the protective immunity. In this study, we identified that aqueous extract of *Artemisia rupestris* L. (AEAR) could be employed as an efficient adjuvant for influenza virus vaccine (V) to enhance immune responses and reduce the antigen doses required for initiating immunity, without compromising the immune response. ICR mice were subcutaneously co-administrated with V combined with different concentrations of AEAR demonstrated that 300 μg AEAR could significantly improve hemagglutination inhibition (HI) and increase IgG antibody titers in serum (*P*<0.05) and the population of CD4^+^CD44^+^ and CD8^+^CD44^+^ (*P*<0.05). Next, 300 μg AEAR combined with different doses of V *in vivo* markedly increased HI and specific IgG antibody level(*P*<0.05). It also significantly increased the amount of CD4^+^ and CD8^+^ T cells, CD4^+^CD44^+^ and CD8^+^CD44^+^ T cells (*P*<0.05), improved lymphocyte proliferation, the secretion of CD4^+^IL-4, CD4^+^IFN-γ and CD8^+^IFN-γ (*P*<0.05), and the killing efficacy of cytotoxic T lymphocyte (CTL) (*P*<0.05). Furthermore, the combination increased the expression of major histocompatibility complex-II (MHC-II) and co-stimulatory molecules including CD40, CD80, and CD86 on dendritic cells (DCs), and downregulated the expression of CD25^+^Foxp3^+^Treg cells (*P*<0.05). No significant difference was observed between high-dose V and low-dose AEAR-V (10-fold lower) vaccination group (*P*>0.05), indicating a 10-fold reduction of antigen required for V vaccine administration. In conclusion, this study demonstrated that AEAR, as an adjuvant for influenza vaccine, could stimulate potent humoral and cellular immune responses and reduce the antigen dose required for effective vaccination, which were mediated by promoting DCs activation and repressing Treg expression.

## Introduction

Influenza, mainly caused by influenza virus, is an acute respiratory disease featuring strong and fast infectivity. It would cause death when complicated with pneumonia in elderly or patients with cardiopulmonary diseases [1, 2]. Currently, the major method on the world for flu precaution and control is vaccination, including injecting inactivated vaccine, attenuated live vaccine, and DNA vaccine [3, 4]. However, due to frequent surface antigen mutation of influenza virus, flu vaccination often cannot meet the need of inoculation dose during flu pandemic [5, 6]. Employing vaccine adjuvant could potentially enhance the effectiveness of vaccine and decrease the volume of vaccine antigen usage, and then more susceptible population would receive vaccination during flu pandemic [7]. Currently, widely used vaccine adjuvants are Al (OH) 3, MF59 and AS03 [8–12]. Alumina gel is the only FDA approved vaccine adjuvant, which can be used in human body. However, it only activates humoral immunity, not cellular immunity [8], which limits the effectiveness, and could not meet the needs during vaccine development.

Traditional Chinese herbal medicine plays a major role in treating diseases [13]. Studies have demonstrated the function of active ingredients of Chinese herbal medicine in immunity regulation, thus they could be the potential sources for developing novel vaccine adjuvants [14]. The polysaccharide group, as the major basis of Chinese herbal medicine, maintains multiple bioactivities such as inoxidizability, anti-tumor effect, and immunity regulation [15].

Several studies have focused on the extraction of polysaccharide from various Chinese herbal medicines, and their effectiveness as vaccine adjuvants have been evaluated. Astragalus polysaccharide, lucid ganoderma, pachymaran, and epimedium have been confirmed to be feasible as vaccine adjuvants [16–20]. *Artemisia rupestris* L. is the perennial herb of rupestris belonging to *Artemisia* (Compositae), which is wildly distributed in Xinjiang (China), middle Asia, and Europe [21]. It is the traditional medicine for Kazakans and Uighurs with antiallergic, antibacterial, and antiviral effects [14]. As a major bioactive component for immune regulation, the polysaccharide of *Artemisia rupestris* L. could be a good candidate for the new vaccine adjuvant.

Our previous studies have demonstrated that AEAR could effectively stimulate the maturation of DCs, and enhance DCs function *in vivo* and *in vitro* [22]. In this study, we investigated the effect of AEAR, as the adjuvant of V, on enhancement of immune responses and antigen sparing in ICR mice, and proposed AEAR as a novel and effective influenza vaccine adjuvant.

## Materials and methods

### Materials

AEAR was prepared in our lab [22]. 2014/2015 seasonal influenza virus split vaccine was purchased commercially from Shenzhen sanofi Pasteur biological products co., LTD (Shenzhen China). Goat anti-mouse peroxidase conjugates (IgG-HRP, IgG_1_-HRP and IgG_2a_-HRP) were purchased from Southern Biotech Inc. USA. Concanavalin A (ConA), lipopolysaccharide (LPS, from Escherichia coli 055:B5) and 3-(4,5-dimethylthiazol-2-yl)-2,5-diphenyltetrazolium bromide (MTT) were from Sigma-Aldrich Co. LLC. USA. Imject Alum Adjuvant (Alum) was from Thermo Scientific Pierce USA. CD11c-FITC, CD3-PE, CD4-APC, CD8a-FITC, CD44-PE, CD25-APC, Foxp3-PE, IFN-γ-PE, IL-4-PE, CD86-PE, CD40-APC, CD80-APC, MHC-II-PE, Cytofix/Cytoperm and Perm/Wash buffer were purchased from BD Bioscience USA. Carboxyfluorescein diacetate succinimidyl ester (CFSE), Treg staining kit were from eBioscience USA. All the other reagents were analytically purified in China.

### Animals and immunization groups

Female ICR mice (6–8 weeks, 18-22g) were purchased from Animal Laboratory Center, Xingjiang Medical University (Urumqi, China). The animal experiment procedures had been approved by the ACUC (Animal Care and Use Committee) of Xinjiang University. Immunization groups is below, Table 1 shows mouse grouping for the screening of AEAR dose. 30 mice were randomly divided into 6 groups (n = 6), and Alum was used as positive control group. Table 2 illustrates mouse grouping for antigen sparing by adding different doses of V with optimal dose of AEAR respectively. 35 mice were randomly divided into 7 groups (n = 6), and subcutaneously co-administered on day 0 and day 14, followed by venous blood collection from mice eyes and serum preparation for further analysis.

**Table 1:** The group of immunization of the screening of AEAR dose.

Groups	Animals	Number	Vaccine
**1**	ICR	5	0.9%NaCl
**2**	ICR	5	V 0.5 μg
**3**	ICR	5	V 0.5 μg + AEAR 100 μg
**4**	ICR	5	V 0.5 μg + AEAR 300 μg
**5**	ICR	5	V 0.5 μg + AEAR 500 μg
**6**	ICR	5	V 0.5 μg + Alum100 μg

**Table 2:** The group of immunization of antigen sparing.

Groups	Animals	Number	Vaccine
**1**	ICR	5	0.9%NaCl
**2**	ICR	5	V 0.05 μg
**3**	ICR	5	V 0.05 μg + AEAR 300 μg
**4**	ICR	5	V 0.1 μg
**5**	ICR	5	V 0.1 μg + AEAR 300 μg
**6**	ICR	5	V 0.5 μg
**7**	ICR	5	V 0.5 μg + AEAR 300 μg

### Analysis of serum antibody responses by ELISA

Indirect ELISA was performed to measure V- specific antibodies levels of IgG, IgG_1_ and IgG_2a_ in serum on day 7, 14, and 21 after first vaccination respectively. V antigen (0.125 μg/mL) was used to coat 96 well culture plate at 4°C overnight, 1% BSA-PBS was used as blocking reagent. Plate was then washed three times by PBS-Tween-20. Diluted serum was added to each well for 1 h at 37°C. Each sample was measured in triplicate. IgG-HRP, IgG_1_-HRP and IgG_2a_-HRP were then added into each well with 1:8000 dilution, and further incubated for 1 h at 37°C. OD_450/655 nm_ was measured with a microtiter plate reader (Bio-Rad, USA) after adding tetramethylbenzidine (TMB) reagent (Sangon Biotech, Shanghai, China) for 10-15min chromogenic reaction [23]. The experimental results were illustrated as antibody titers or OD value.

### HI analysis

V antigen was diluted with PBS and 100 μL was added to blood clot plate. 50 μL guinea pig red blood cell suspensions was then added into each well for 30 min at room temperature, four hemagglutination unit antigen was made for HI test. Double diluted serum after immunization and red blood cell suspensions were incubated with four hemagglutination unit antigens for 30 min at room temperature for further hemagglutination inhibition measurement. The highest dilution of serum that prevented hemagglutination was considered as the HI titer [24].

### Lymphocytes proliferation assay

Spleen cell suspension was obtained from mice on day 21 after first vaccination. 5x10^6^ spleen cells were plated into each well (96 well plates), ConA (3.5 μg/mL) or LPS (5 μg/mL) were added into each well and incubated for 48 h at 37°C. 100 μL DMSO were then added to each well for OD570/630 nm measurement after another 4 h culturing with the addition of 20 μL MTT solution (5 g/L). SI (stimulation index) was calculated based on the following formula: SI = (stimulated well OD—medium OD)/ (non-stimulated OD—medium OD) [25].

### Cell surface markers staining

Cell surface markers were stained and analyzed by FACS [26]. First, single cell suspension (1x10^6^ cells) from splenocytes in mice was obtained, and washed using 0.5% FBS-PBS. After addition of antibodies, cells were cultured for 30 min avoiding the light. After washing with PBS, cells were then detected by FACS. Data were analyzed with FlowJo.

### Analysis of cytokines and Treg cells

FACS was performed for intracellular cytokines staining. Generally, 2x10^6^ cells from splenocytes in mice added into 24-well cell culture plate were further stimulated by antigens for 4 h. Golgistop (BD Biosciences, USA) was then added and cultured overnight. Cells were then collected for surface markers staining and/or washed for intracellular cytokines staining. Cytofix/Cytoperm was added and incubated for 30 min at 4°C to lyse cells. Cells were then washed by Perm/Wash buffer, incubated with PE-anti-IL-4 mAb (BD Biosciences, USA), PE-anti-IFN- γ mAb (BD Biosciences, USA), and PE-anti-Foxp3 mAb respectively for 20 min in the dark, and analyzed by FACS [27, 28].

### CTL analysis *in vivo*

A detailed description can be found in references [29]. In brief, we divided Naïve mouse spleen cells into two identical groups, 15 μg V was added into one group, the other group was control without V for 4 h at 37°C. Cells treated with V were incubated with 10 μM (CFSE^high^), while untreated cells were incubated with only 1 μM (CFSE^low^) for 15 min. We further mixed the two groups’ cells with equivalent volume and injected totally 2x10^7^ cells into the immunized mice on day 21 after first vaccination through tail vein. After 4h injection, spleen cells were obtained and FACS analyses were performed. Specific lysis was calculated using the following formula: ratio = percentage CFSE^low^/percentage CFSE^high^. Percentage specific lysis = [1 − (ratio unprimed/ratio primed) × 100].

### Statistical analysis

All data were presented as mean ± SD. Prism 5.0 was used for data analysis. To compare between groups, one-way ANOVA and Tukey's Multiple-Comparison Test, or Student’s t-test were used. * P<0.05 indicates a significant difference; ** *P*<0.01 indicates that difference is extremely significant.

## Results

### The effect of different AEAR doses on HI titer and serum IgG

**Fig 1:**
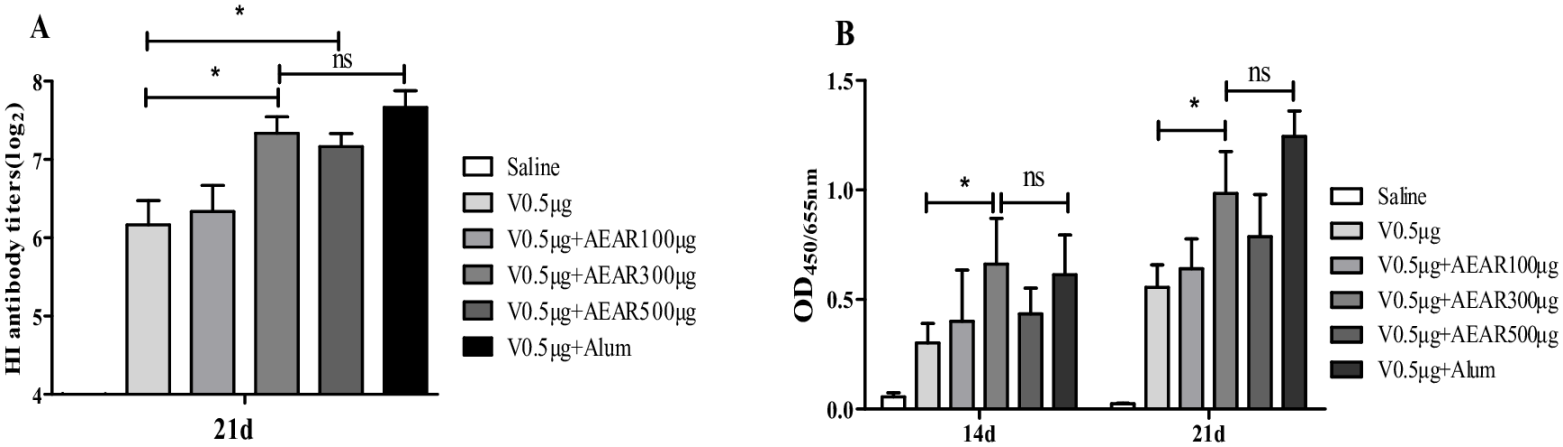
Detection of HI antibody titer and serum IgG antibody. (A) The titer of HI antibody against V antigen on day 21 after first vaccination with 0.5 μg V mixed with 100 μg, 300 μg, and 500 μg AEAR adjuvants respectively. (B) Detection of IgG by indirect ELISA in serum on day 14 and 21 after first vaccination. Data were expressed as means±SE (n = 6). The results were representatives of three independent experiments. * *P*<0.05, ^ns^ *P*>0.05.

In order to identify the optimal AEAR doses, 100 μg (low-dose group), 300 μg (medium-dose group), and 500 μg (high-dose group) of AEAR combined with 0.5 μg V were used respectively to immunize ICR mice. Alum was used as the positive control. On day 21 after first vaccination, the titer of HI antibody in serum was measured (Fig 1A). As shown in Fig 1A, the titer of HI antibody was significantly higher in AEAR medium-dose and high-dose groups than in V-only group (*P*<0.05), and the difference between AEAR medium-dose group and Alum group was not significant (*P*>0.05). Indirect ELISA was performed to measure the IgG antibody level in serum on day 14 and 21 after first vaccination (Fig 1B). We found that the IgG antibody level was significantly higher in AEAR medium-dose than in V-only group (*P*<0.05), and the difference between AEAR medium-dose group and Alum group was not significant (*P*>0.05). These findings suggested that AEAR could enhance serum HI titer and antibody level.

### The effect of different AEAR doses on CD44^+^ T cell

**Fig 2:**
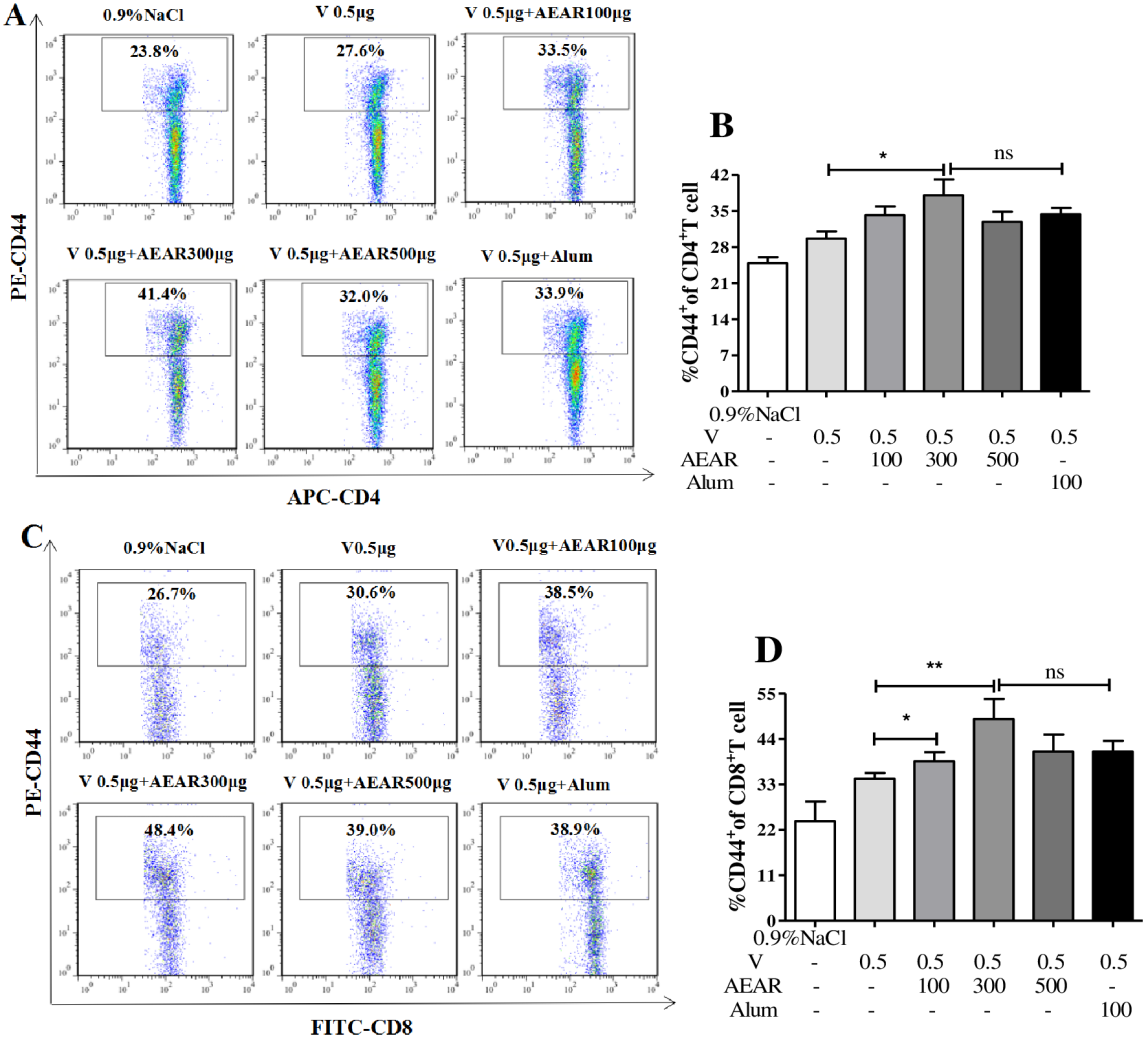
Analysis of CD4^+^CD44^+^ T cells and CD8^+^CD44^+^ T cells. Mice in the AEAR treatment groups had an increased proportion of CD44^+^CD4^+^ T cells (A-B) and CD44^+^CD8^+^ T cells (C-D) in spleen compared with the V group measured by FACS. Data were expressed as means±SE (n = 6). The results were representatives of three independent experiments. * *P*<0.05, ** *P*<0.01, ^ns^ *P*>0.05.

On day 7 after second vaccination, the amount of CD4^+^CD44^+^ and CD8^+^CD44^+^ T cells in spleen was measured by FACS (Fig 2A–2D). The percentage of CD4^+^CD44^+^T cells was higher compared with V-only group (*P*<0.05). There was no significant difference (P>0.05) when compared with Alum group. The amount of CD8^+^CD44^+^T cells in both AEAR low-dose and medium-dose group was higher than in V-only group (*P*<0.05), there was no significant difference when compared with Alum group (*P*>0.05). These results indicated that AEAR could effectively improve antigen-specific T cell immune responses.

### Measurement of serum HI and IgG antibody of antigen sparing

**Fig 3:**
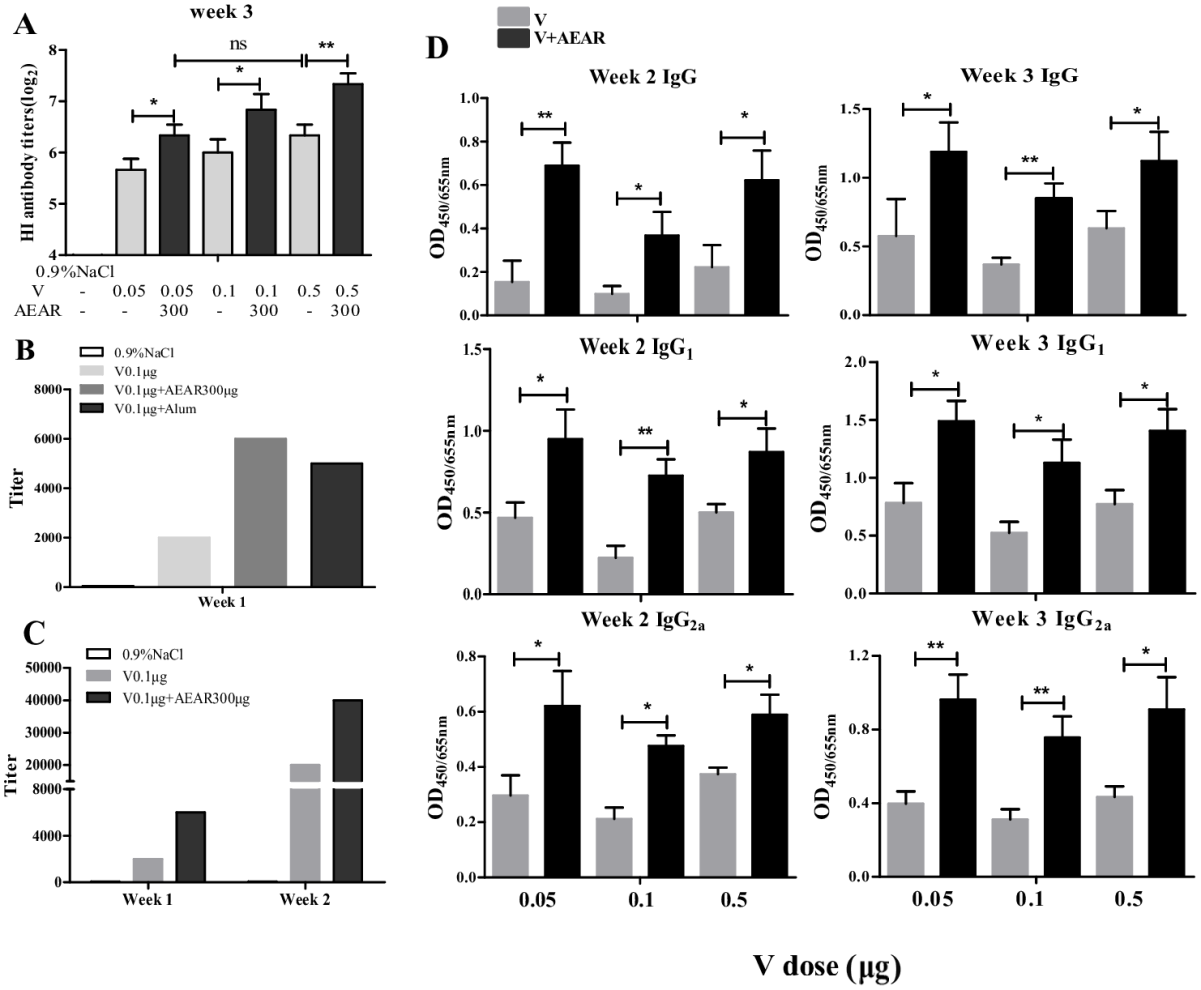
Detection of HI antibody titer and serum IgG, IgG_1_, IgG_2a_ of antigen sparing. (A) HI antibody titer against different doses of V antigen after vaccination of V 0.05 μg, V 0.1μg, and V 0.5 μg mixed with 300 μg of AEAR on day 21 after first vaccination. (B) Detection of IgG titer in serum by indirect ELISA on day 7 after first immunization. (C) IgG titer in serum on day 7 and 14 after first immunization. (D) Detection of antibody IgG, IgG_1_, IgG_2a_ in serum on day 14 and 21 after first immunization. Data were expressed as means SE (n = 6). The results were representatives of three independent experiments. * *P*<0.05, ** *P*<0.01, ns *P*>0.05.

In order to further identify if ARAR can reduce V antigen dose required for immunization, we vaccinated the ICR mice with the optimized 300 μg AEAR together with 0.05 μg (low-dose), 0.1 μg (medium-dose), or 0.5 μg (high-dose) V respectively. HI test was next performed to measure the HI antibody titer on day 21 after first vaccination (Fig 3A). The HI titers in all three groups were higher compared with V-only group (*P*<0.05). No significant difference can be observed between AEAR-V-low-dose group and V-only-high-dose group (*P*>0.05), which indicated that the antigen dose required for immunization could be reduced by 10 folds.

To identify if IgG antibody can be produced in early phase after vaccination, we measured the titer of IgG antibody on day 7 after first vaccination (Fig 3B), IgG titer in AEAR-V-medium-dose group was 3 times higher than in V-only group, which was higher than in Alum group. IgG titer was also increased in AEAR-V-medium-dose group on day 7 and 14 after first vaccination (Fig 3C). Expression level of IgG, IgG_1_, and IgG_2a_ in all (low, medium and high dose) groups are dramatically higher than in V-only group (*P*<0.01). There was no significant difference between AEAR-V-low-dose group and V-only-high-dose group (*P*>0.05). It indicated that antigen dose required for immunization can be reduced by 10 folds (Fig 3D).

### Lymphocyte proliferation assay

**Fig 4:**
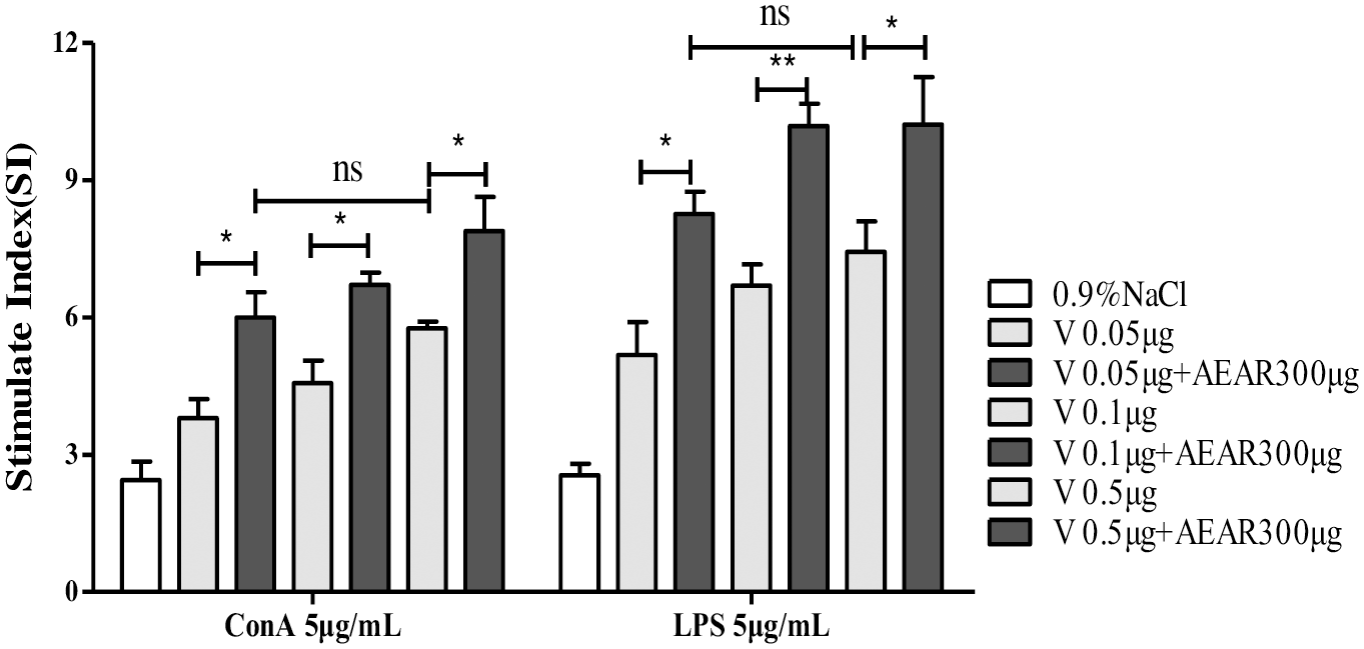
Splenic lymphocyte proliferation assay. *In vitro* spleen lymphocyte proliferation assays from mice treated with AEAR 300μg combined with different doses respectively on day 21 after first vaccination. Data were expressed as means±SE (n = 6). The results were representatives of three independent experiments. * *P*<0.05, ** *P*<0.01, ^ns^ *P*>0.05.

To further determine the effect of AEAR on cellular immune response, Lymphocyte proliferation assay was performed. The proliferation of lymphocytes from mice was measured by MTT on day 21 after first vaccination. First, the suspension of spleen cells in mice was prepared. Then, SI was measured after adding ConA or LPS (Fig 4). Fig 4 showed that the proliferation of lymphocytes induced by ConA or LPS were increased in all AEAR-V-low, medium and high groups(*P*<0.05). No significant difference was detected between AEAR-V-low-dose group and V-only-high-dose group (*P*>0.05). It indicated that antigen dose required for immunization could be reduced by 10 folds.

### T cell population analysis

**Fig 5:**
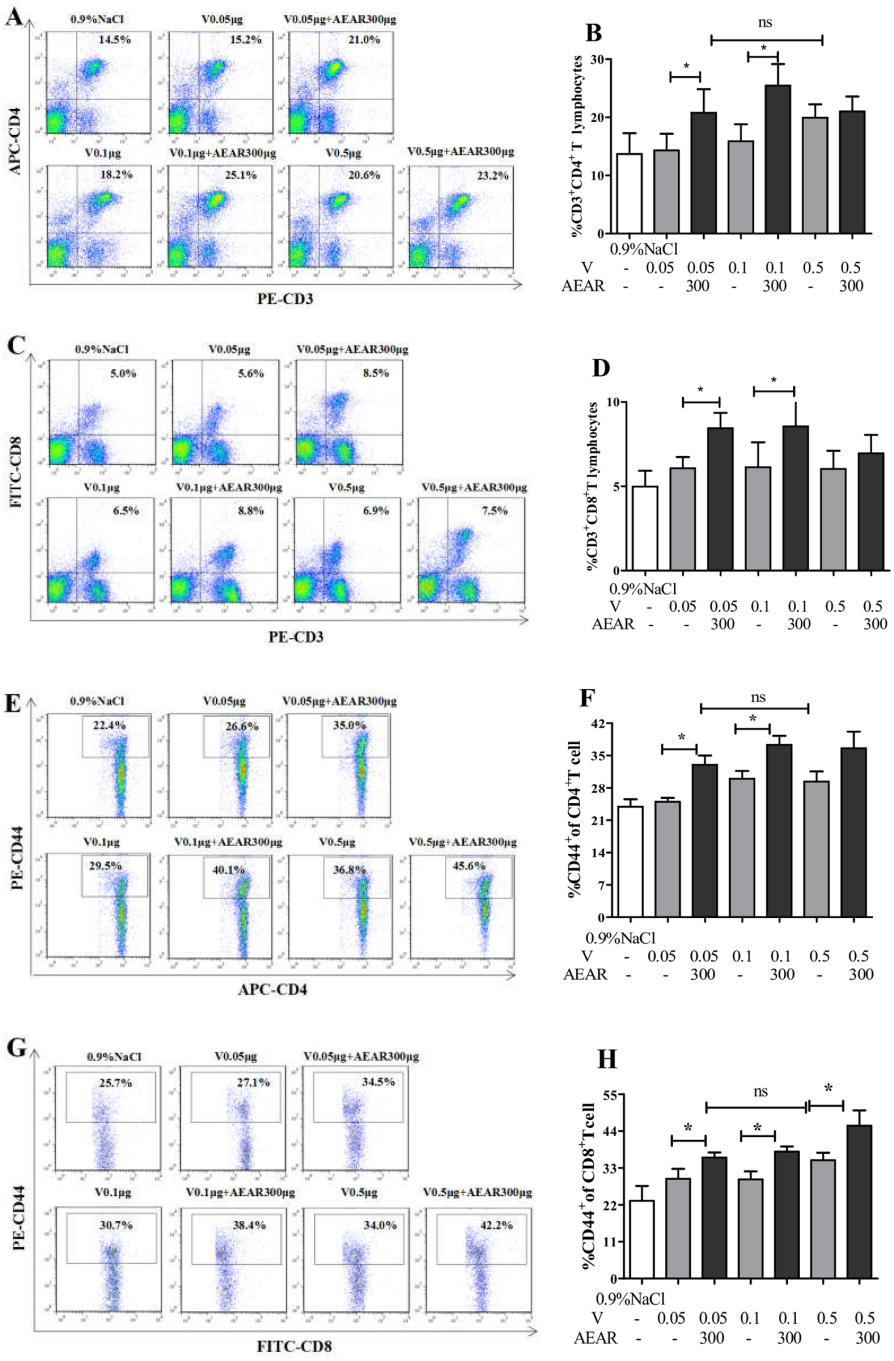
Detection of the percentages of T lymphocyte subsets by FACS. The lymphocyte subpopulation of CD3^+^CD4^+^(A-B), CD3^+^CD8^+^ T cells(C-D), CD4^+^CD44^+^ T cells (E-F), and CD8^+^CD44^+^ T cells(G-H) by FACS. The spleen lymphocytes were collected on day 21 after first vaccination from the mice immunized with AEAR and the different doses of V for detecting T lymphocyte subsets. Data were expressed as means±SE (n = 6). The results were representatives of three independent experiments. * *P*<0.05, ** *P*<0.01, ^ns^ *P*>0.05.

T cell response plays a major role in immune response against influenza virus. Using FACS, we measured the percentages of CD4^+^, CD8^+^, CD4^+^CD44^+^, CD8^+^CD44^+^ T cells in spleen on day 21 after first vaccination (Fig 5). There were more CD4^+^ and CD8^+^ T cells in AEAR-V-low-dose and medium-dose groups compared with the V-only group (*P*<0.05) (Fig 5A–5D). The percentage of CD4^+^CD44^+^ and CD8^+^CD44^+^ T cells in AEAR-V-low-dose and medium-dose groups are also significantly higher compared with V-only group (*P*<0.05). No significant different can be observed between AEAR-V-low-dose group and V-only-high-dose group (*P*>0.05). Antigen dose required for immunizations is reduced by 10 folds (*P*<0.05).

### Cytokine expression analysis

**Fig 6:**
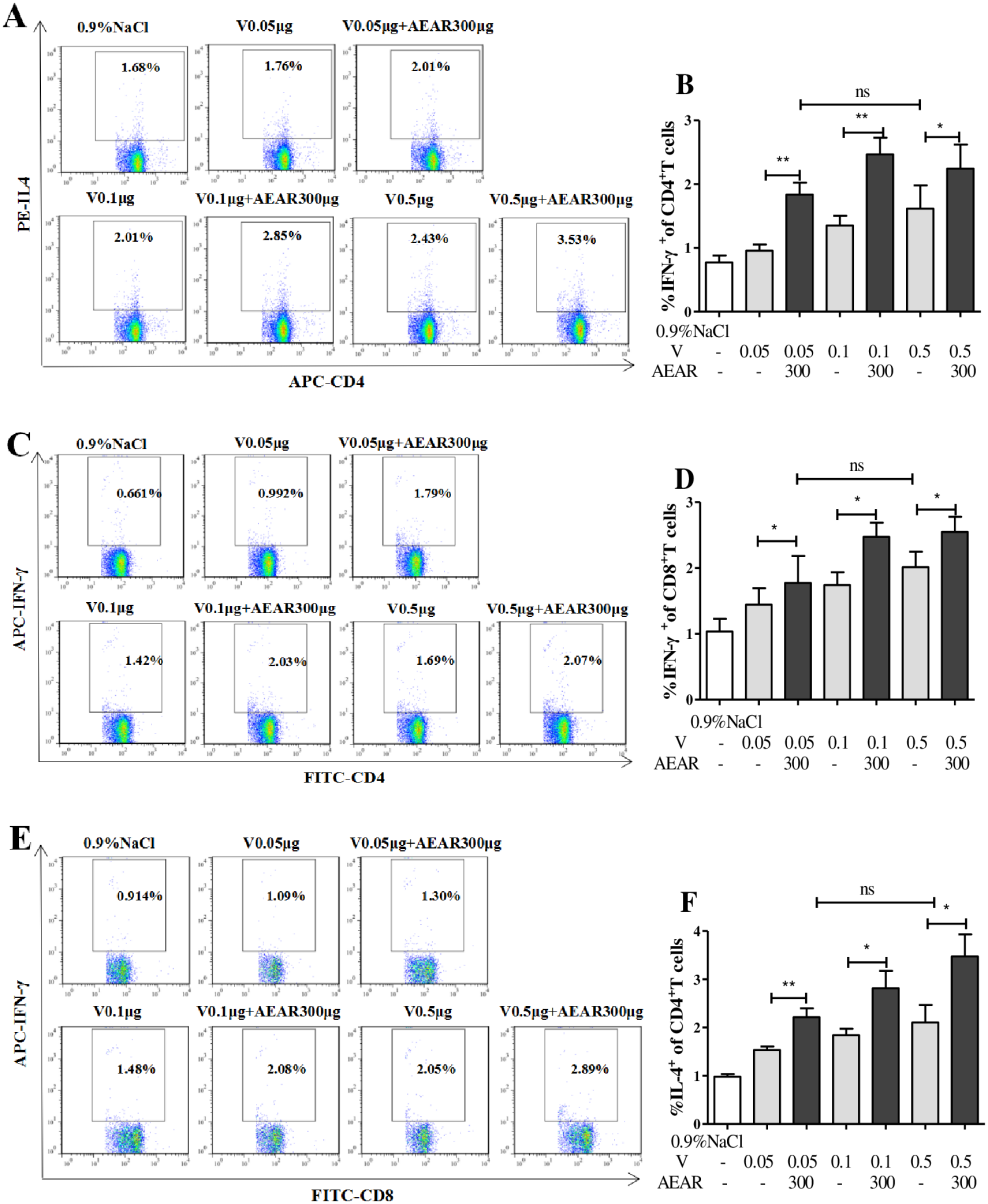
Analysis of antigen-specific cytokine production in T cells by FACS. T cells isolated from the spleen of ICR mice on day 7 after second immunization. The percentages of IL-4 (A-B) in CD4^+^, IFN-γ(C-D) in CD4^+^, IFN-γ(E-F) in CD8^+^ were shown. Data were expressed as means±SE (n = 6). The results were representatives of three independent experiments. * *P*<0.05, ** *P*<0.01, ^ns^ *P*>0.05.

Cytokines play an important role in immune response. We measured the expression of cytokines in spleen cells by FACS on day 7 after second vaccination (Fig 6). The secreted CD4^+^IL-4 in AEAR-V-low-dose, medium-dose, and high-dose groups were significantly higher than in V-only group (*P*<0.05) (Fig 6A and 6B). The expression of CD4^+^IFN-γ and CD8^+^IFN-γ in all three groups were higher than in V-only group (*P*<0.05) (Fig 6C–6F). No significant different can be observed between AEAR-V-low-dose group and V-only-high-dose group (*P*>0.05). These results indicated that AEAR could stimulate both humoral and cellular immune response, especially T-cell mediated immune response.

### DCs activation and Treg expression

**Fig 7:**
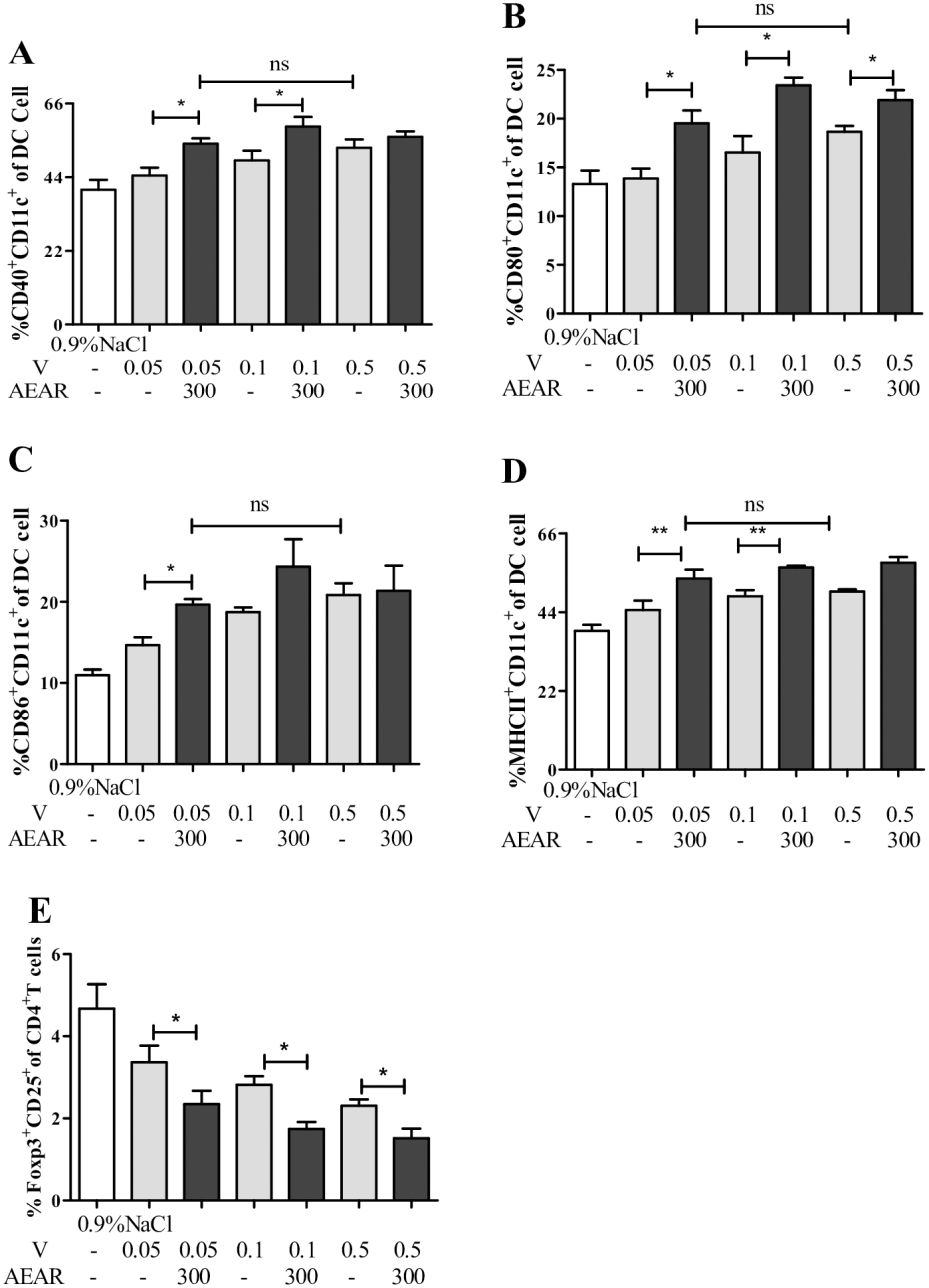
Analysis of DCs maturation and the expression of Tregs by FACS. Total splenocytes were isolated from the spleens of ICR mice on day 3 or 21 after first vaccination.(A-E) Double staining for CD11c^+^CD40^+^), CD11c^+^CD80^+^, CD11c^+^CD86^+^ and CD11c^+^MHC-II^+^ was performed. The percentages of CD11c^+^CD40^+^, CD11c^+^CD80^+^, CD11c^+^ CD86^+^, and CD11c^+^ MHC-II^+^ cells in the total cells are shown in (A), (B), (C) and (D) respectively. E) Treg expressions were analyzed by FACS. Splenocytes were isolated from ICR mice on day 3 after The expressions of CD4^+^CD25^+^Foxp3^+^cells were determined by FACS (I-J). Data were expressed as means±SE (n = 6). The results were representatives of three independent experiments.* *P*<0.05, ** *P*<0.01, ^ns^ *P*>0.05.

It is well known that the activation of DCs and the expression of Tregs are important in initiating specific immune response and immunoregulation. To investigate the mechanisms of AEAR enhancing the immune response to V and antigen sparing, we determined the expression of surface molecules CD40, CD80, CD86 and MHC-II on DCs from splenocytes in mice on day 3 after first vaccination (Fig 7A–7D), and the expression of Tregs on day 7 after second vaccination (Fig 7E). The expression of all four surface molecules on DCs in AEAR-V-low-dose and medium-dose groups were higher compared to V-only group (*P*<0.05). There was no significant difference between AEAR-V-low-dose group and V-only-high-dose group (*P*>0.05). The expression of CD25^+^Foxp3^+^ Treg in AEAR-V-low-dose, medium-dose and high-dose group were remarkably lower than in V-only group (*P*<0.05), which indicates that AEAR could stimulate the maturation of DCs and downregulate the expression of Tregs.

### CTL analysis

**Fig 8:**
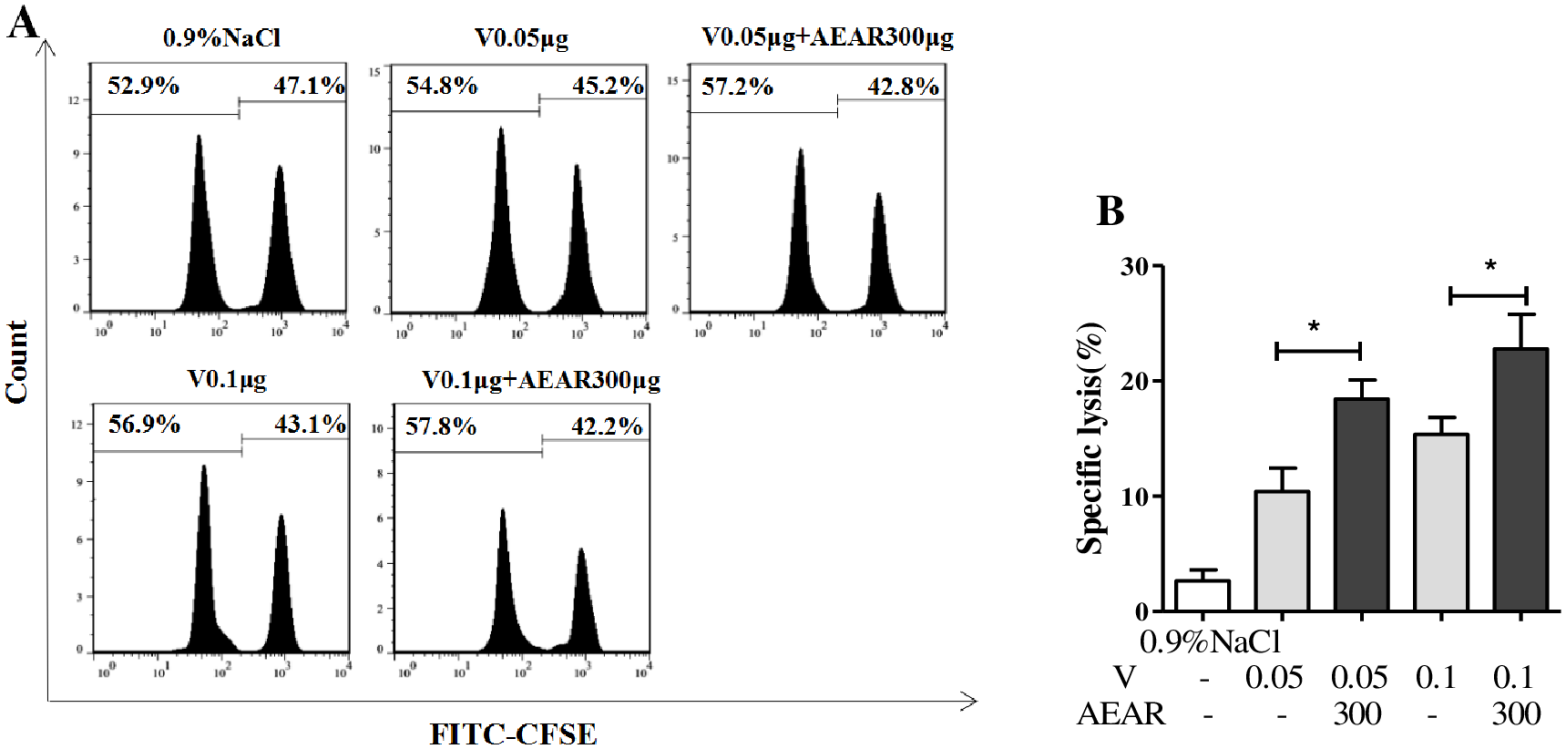
CTL analysis in mice spleen. (A) CFSE-labelled target cells at a ratio of 1:1 (CFSE^high^ to CFSE^low^) from naive ICR mice were injected into syngeneic mice on day 21 after first vaccination. 4 h later, the mice were euthanized, and the CFSE-labelled cells were analyzed for ratio changes in the CFSE^high^ and CFSE^low^ target cell populations. (B) The percentage of specific lysis was determined for each group as described in methods. Data were expressed as means±SE (n = 5). The results were representatives of three independent experiments. * *P*<0.05.

CTL plays an important role in immune response against influenza virus. To investigate the CTL response in mice after vaccination, the CTL specific killing rate was measured by FACS on day 21 after first vaccination (Fig 8A and 8B). As shown in Fig 8A and 8B, the CTL killing rate in AEAR-V-low-dose and medium-dose groups were significantly higher compared to V-only group (*P*<0.05), indicating that AEAR could stimulate CTL response, which promoted the elimination of influenza viruses.

## Discussion

The effective components of Chinese herbal medicine have various pharmacological functions including antivirus and anti-inflammatory, which can be used as a treatment for autoimmune diseases [30,31]. Especially, their immunoregulation function makes it a hot topic in the development of novel, safe, and efficient vaccine adjuvant [32]. With the development of modern isolation technology, more bioactive components have been determined and isolated, such as polysaccharides, saponin, and flavone, which have been employed as adjuvants to activate antigen-presenting cells (APCs), promote cytokines production, and enhance antiviral and anti-inflammatory immune responses [33,34]. Previous studies indicated that plant polysaccharides, as adjuvants, could activate T cells, B cells, and other immune cells, also activate complement, and induce interferon and cytokines production to boost immune responses [35].

It is very important to influenza vaccines that elicit robust humoral and cellular immune responses. One of these strategies of improving vaccine-mediated protection against influenza is to add adjuvants into the vaccine. Adjuvants, by promoting antigen presentation, activation and maturation of dendritic cells and production of inflammatory cytokines, can drive the desired humoral and T-cell-mediated responses to influenza vaccines.

In this study, we investigated the role of AEAR as a V adjuvant in improving immune responses and reducing antigen dose needed for effective V vaccination. Our results demonstrated that ideal immune effect was achieved with combinations of appropriate concentration of AEAR and V antigen. We first investigated the effect of different concentrations of AEAR on immune responses including HI and IgG antibody levels in serum [36]. Our results showed that HI titer, IgG level, and CD44^+^ T cell responses were significantly improved by AEAR, and the optimal doses for vaccination were 300 μg in our experimental settings. We further investigated the role of AEAR in the reduction of antigen dose required for immunization by using the optimal dose of AEAR (300 μg) combined with low, medium, or high doses of V for vaccination respectively. HI and IgG antibody level results demonstrated that 300 μg AEAR remarkably boosted humoral immune responses even with 10-fold lower dose of V. IgG antibody levels in mice on day 7 after immunization confirmed that AEAR could be used as a V adjuvant to rapidly stimulate high antibody levels in the serum, which would provide early prevention from influenza infection.

The efficacy of influenza vaccine is associated with stimulation of cell-mediated and cytotoxic T-lymphocyte responses. Cell-mediated immunity, an immune response mediated mainly by T cells, plays an important role in the prevention and control of influenza virus [37]. T cells have many subgroups characterized by their surface markers. For example, CD4^+^ T cells can promote the maturation of B cells or facilitate the antigen presentation of APCs, while CD8^+^ T cells are capable of directly killing the target cells [38]. In this study, we analyzed the proliferation of lymphocytes, T-cell subsets, and CTL efficacy to determine if AEAR-V induced T-cell responses. Our results showed that AEAR-V significantly enhanced ConA-stimulated T-lymphocyte proliferation and LPS-induced B-cell proliferation. Moreover, AEAR-V notably boosted the number of CD4^+^, CD8^+^, CD4^+^CD44^+^, and CD8^+^CD44^+^ T cells, improved CTL efficacy, and achieved a 10-fold reduction of the antigen dose required for immunization without compromising the immune response. These results suggested that AEAR could increase V-induced T-cell immunity via affecting T lymphocytes to control influenza virus infection.

During the antigen recognition and activation of immune responses, cytokines stimulate the proliferation of immune cells, IFN-γ induces CTL activation to initiate the elimination of influenza virus [39–41], IL-4 can stimulate the proliferation of B-lymphocytes and antibodies production to neutralize influenza virus [42]. Our data showed that AEAR-V significantly increased IL-4 and IFN-γ expression, indicating that as a V adjuvant, AEAR induces potent humoral and cellular immunity, especially the T-cell mediated immune responses.

The maturation and activation of DCs is a key step to initiate the antigen-specific immune responses. Treg cells are a subgroup of T lymphocytes with immunosuppression and regulation function [43]. To understand the mechanism of AEAR enhancing V-induced immunity, we investigated the maturation of DCs and the number of Treg cells in spleen in immunized mice. It demonstrated that AEAR-V boosted the expression of CD40, CD80, CD86, and MHC-II on DCs, inhibited Treg frequency, which indicated that AEAR, as a V adjuvant, could improve immune responses via promoting DCs maturation and repressing Treg frequency.

In conclusion, AEAR, as an influenza virus vaccine adjuvant, can induce specific humoral and cellular immunity, especially the T-cell mediated immune responses, and reduce the antigen dose required to initiate protective immunity, by promoting the maturation of DCs and downregulating Treg expression. Therefore, AEAR can be developed as an efficient commercial adjuvant for influenza virus vaccine. Furthermore, this study provides a novel and efficient strategy to address the potential shortage of vaccine supply during flu pandemic.
